# Electrostatic Self-Assembled Chitosan-Pectin Nano- and Microparticles for Insulin Delivery

**DOI:** 10.3390/molecules22101707

**Published:** 2017-10-12

**Authors:** Vinicius B. V. Maciel, Cristiana M. P. Yoshida, Susana M. S. S. Pereira, Francisco M. Goycoolea, Telma T. Franco

**Affiliations:** 1 Faculty of Animal Science and Food Engineering, USP—University of São Paulo, Av. Duque de Caxias Norte, 225, Pirassununga CEP 13635-900, São Paulo, Brazil; viniciusbvm@usp.br; 2 Department of Exact and Earth Science, UNIFESP—Federal University of São Paulo, Rua São Nicolau, 210, Diadema CEP 09913-030, São Paulo, Brazil; 3 Institut für Biologie und Biotechnologie der Pflanzen, Westfälische Wilhelms—Universität Münster, Schlossgarten 3, 48149 Münster, Germany; ssoar_01@uni-muenster.de; 4 School of Food Science and Nutrition, University of Leeds, Leeds LS2 9JT, UK; 5 School of Chemical Engineering, UNICAMP—State University of Campinas, Av. Albert Einstein, 500, Campinas CEP 13083-852, São Paulo, Brazil; franco@feq.unicamp.br

**Keywords:** chitosan, pectin, microparticles, nanoparticles, insulin, Caco-2 cells

## Abstract

A polyelectrolyte complex system of chitosan-pectin nano- and microparticles was developed to encapsulate the hormone insulin. The aim of this work was to obtain small particles for oral insulin delivery without chemical crosslinkers based on natural and biodegradable polysaccharides. The nano- and microparticles were developed using chitosans (with different degrees of acetylation: 15.0% and 28.8%) and pectin solutions at various charge ratios (n^+^/n^−^ given by the chitosan/pectin mass ratio) and total charge. Nano- and microparticles were characterized regarding particle size, zeta potential, production yield, encapsulation efficiency, stability in different media, transmission electron microscopy and cytotoxicity assays using Caco-2 cells. The insulin release was evaluated in vitro in simulated gastric and intestinal media. Small-sized particles (~240–~1900 nm) with a maximum production yield of ~34.0% were obtained. The highest encapsulation efficiency (~62.0%) of the system was observed at a charge ratio (n^+^/n^−^) 5.00. The system was stable in various media, particularly in simulated gastric fluid (pH 1.2). Transmission electron microscopy (TEM) analysis showed spherical shape particles when insulin was added to the system. In simulated intestinal fluid (pH 6.8), controlled insulin release occurred over 2 h. In vitro tests indicated that the proposed system presents potential as a drug delivery for oral administration of bioactive peptides.

## 1. Introduction

Chitosans are a family of polycationic aminopolysaccharides obtained from chemical or enzymatic modification of chitin. Among the key properties of chitosan, the capacity to form self-assembled electrostatic complexes with oppositely charged macromolecules, including polysaccharides, nucleic acids or proteins is one the most important. Macromolecular complexation by electrostatic self-assembly between chitosan and oppositely charged polymers can lead to highly stable colloidal systems [1,2]. Maciel et al. developed a polyelectrolyte complex matrix formed between chitosan and pectin to entrap a bioactive and hydrophilic compound (anthocyanin) that acts as a useful pH indicator device [3]. Some characteristics of the particles can be modified to increase the encapsulation efficiency (EE) of bioactive compound in the nanoparticles [4] such as the hydrodynamic diameter, morphology, zeta-potential (ζ-potential), and varying the ratio of either polymer or ionic crosslinker. Among the advocated advantages of nanostructured particles are ability to form a material resistant to acidic denaturation and enzymatic degradation, prolonging their residence time in the small intestine and hence, modulating the kinetics of release and delivery of the bioactive payload [5,6]. Rao and Geckeler [7], and Kreuter [8] have affirmed that nanoparticles could be defined as solid spherical particles usually ranging between 10–1000 nm and, that they are more suitable for drug delivery systems than microparticles. In this work, we have harnessed the capacity of electrostatic self-assembly of chitosan and pectin to form biocompatible macromolecular complexes that can be used in drug delivery. Pectin is a natural, low toxicity and polyanionic polysaccharide extracted from the cell walls of most plants, such as apples, oranges and pears. As chitosan does, pectin also exhibits material and bioactive properties. Chemically, it is a branched heteropolysaccharides, which consist predominantly of linear chains of partially methyl-esterified (1,4)-α-D-galacturonic acid residues [9].

The International Diabetic Federation reported that 366 million people were affected by diabetes in 2011 and estimated that this number will increase to 552 million in 2030 [10,11]. Diabetes is one of the most lethal diseases due to its high prevalence and secondary effects. The World Health Organization [12] reported that it is responsible for thousands of deaths per year worldwide. Insulin, a protein composed by 51 amino acid residues, is the most common and effective drug used to control diabetes [13,14]. Peptides and proteins have been used as biopharmaceuticals due to their high activity, specificity and effectiveness compared to conventional drugs [10]. However, suitable formulations intended for oral administration based on proteins are still under development. During their transit through the gastrointestinal tract, drug molecules encounter several metabolic and physicochemical barriers. These include the harsh acidic environment of the stomach, extensive enzymatic degradation by various proteolytic enzymes, poor intestinal absorption, mucus clearance and overcoming the epithelial cell barrier [15]. The combination of these obstacles limits the systemic absorption of drugs and consequently, their therapeutic functions [16]. The use of oral formulations to deliver insulin with high bioavailability has been a subject of intense research efforts However, up to now, no successful formulations showing high insulin bioavailability have been marketed.

The use of biocompatible and biodegradable polymeric nano- and microparticles have been described as a promising strategy for oral administration of proteins such as insulin and peptides [17], providing a stable environment for the encapsulated drug [18]. An increasing number of studies have shown that polymeric nano- and microparticles are effective as drug carriers. Mukhopadhyay et al. developed self-assembled chitosan–insulin nanoparticles for oral insulin delivery and evaluated their efficiency using an in vivo diabetic model [16]. Al-Azi et al. [19] investigated insulin encapsulation, as a model drug, using high molar mass pectin–chitosan coacervates. Mukhopadhyay et al. developed chitosan–alginate nanoparticles for oral insulin administration by ionotropic pre-gelation of alginate to entrap insulin within the inner core, followed by polyelectrolyte complexation with chitosan [20]. Andreani et al. found evidence that silica nanoparticles coated with hydrophilic polymers (chitosan, sodium alginate and polyethylene glycol) can function as mucoadhesive carriers for oral insulin administration [21]. Further, Chronopoulou et al. developed an optimised drug delivery carrier (chlorhexidine) for oral mucosa use, able to deliver bioactive molecules in situ, using chitosan-based nanoparticles [22]. In turn, Zhang et al. prepared a novel polyelectrolyte complex based on cationic chitosan and anionic poly(2-acrylamide-2-methyl-propanesulfonic acid) using a polymer–monomer pair reaction system to form nanoparticles [23]. Our study complements previous works, by expanding our understanding on biopolymer-based carriers intended for oral insulin delivery. To this end, we developed insulin-loaded nano- and microparticles based on pectin and chitosan of low molar mass and two distinct degrees of acetylation (DA). We then studied the influence of the composition and DA of chitosan on the size distribution, ζ-potential, production yield, stability in different biological media, insulin EE, morphology by transmission electron microscopy (TEM), cytotoxicity by Caco-2 cells and insulin in vitro release.

## 2. Results and Discussion

### 2.1. Chitosan and Pectin Characterisation

The degrees of acetylation of chitosan (Heppe medical chitosan) HMC^+^ 85/5 and HMC^+^ 70/5 were 15.0% and 28.8%, respectively. The intrinsic viscosity ([*η*]) and molar mass of HMC^+^ 15 were 179.4 mL·g^−1^ and 2.74 × 10^4^ g·mol^−1^, respectively, and 124.2 mL·g^−1^ and 1.75 × 10^4^ g·mol^−1^, respectively, for HMC^+^ 70/5. The degree of esterification (DE) of pectin was 68.2%, being classified as a highly methoxylated pectin and the [*η*] and molar mass of this polysaccharide were 429.5 mL·g^−1^ and 1.01 × 10^5^ g·mol^−1^, respectively. The DA of chitosan, DE of pectin, as well as the polymer molar mass, are known to be important polymer characteristics to consider when forming nano- and microparticles [24,25,26].

### 2.2. Nano- and Microparticles Characterisation

Preliminary tests were developed to evaluate the nano- and microparticle formation over a wide range of charge ratio (n^+^/n^−^) (varying from 0.10 to 5.00). The optimal formulations were chosen, namely with excess pectin charge and excess chitosan charge. The formation of nano- and microparticles was verified visually from the solution turbidity and the absence of polymeric agglomeration, under all the tested conditions. These included various charge ratios (n^+^/n^−^), total charge (1.0 or 2.0 × 10^−6^ M), pH (4.0, 5.0, 5.5 and without pH adjustment) and DA of chitosan (15.0% and 28.8%). Particles were formed using chitosan with DA of 15.0% and without adjusting the pH at charge ratios (n^+^/n^−^) 2.00, 4.00 and 5.00. Using chitosan with 28.8% DA, the best charge ratios (n^+^/n^−^) to form particles were 1.33, 2.00, 4.00 and 5.00.

**Figure 1:**
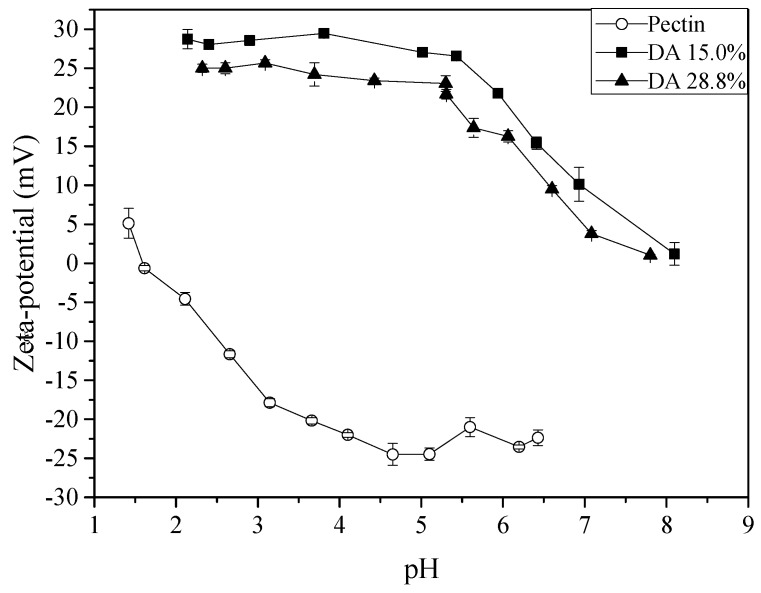
Variation of the ζ-potential (zeta-potential) with pH for chitosan and pectin solutions prepared in 100 mM NaCl (as in legend) at 25 °C.

The behaviour of each polyelectrolyte solutions and the best pH range to obtain further nano- and microparticles were assessed for chitosan (DA 15.0% and 28.8%) and pectin (Figure 1). To this end, it was measured the pH-dependence of the ζ-potential of aqueous solutions of both polyelectrolytes (chitosan and pectin) in the pH range evaluated (1.0–8.0).

It is noted in Figure 1 that no major change in ζ-potential occurred in the pH range of ~2.0 to ~5.0. Beyond pH 5.0, the ζ-potential decreased from ~+27.0 to ~+7.0 mV for both chitosan solutions. These results are consistent with the reported intrinsic p*K*_o_ of chitosan (~6.1) [27,28]. At pH < p*K*_o_, the amino groups of chitosan are predominantly protonated, which confers the molecule a polycationic character. In turn, for pectin the surface net charge remained negative (ζ-potential = ~−25.0 mV) at pH > 4.5. However, at pH < 4.5, the ζ-potential values decreased monotonically until nearly reaching zero at pH 1.5. The ζ-potential is defined as the electrical potential at the hydrodynamic shear plane with respect to that of the solvent [29]. In our work, we have measured ζ-potential the only as an indication of the charge state of the polyelectrolytes at the given working pH. Measurements of ζ-potential as a function of ionic strength have been used by Abodinar et al., to estimate the conformation and chain flexibility of polyelectrolytes in dilute solution and their dependence on ionic strength [30]. Although the ζ-potential parameter offers a close picture of the net degree of ionization of the polyelectrolyte in solution at a given pH, the fact that it is sensitive to the structural and conformational aspects of the polyelectrolyte, it cannot be regarded as a surrogate of potentiometric nor conductimetric measurements.

The ζ-potential pH-dependence observed for pectin agrees with the reported p*K*_a_ of 3.5–4.5 [31]. At pH < 4.5, the anionic character of pectin is weakened due to the dissociation of the carboxylic groups of the pectin structure [32]. In preliminary tests, we observed that when the chitosan–pectin complexes were formed at pH ~5.0, agglomeration occurred. Coimbra et al. have suggested that the available charges at low pH could decrease the agglomeration process of the polymer solution [26].

### 2.3. Zeta-Potential and Particle Size Distribution Analysis

**Figure 2:**
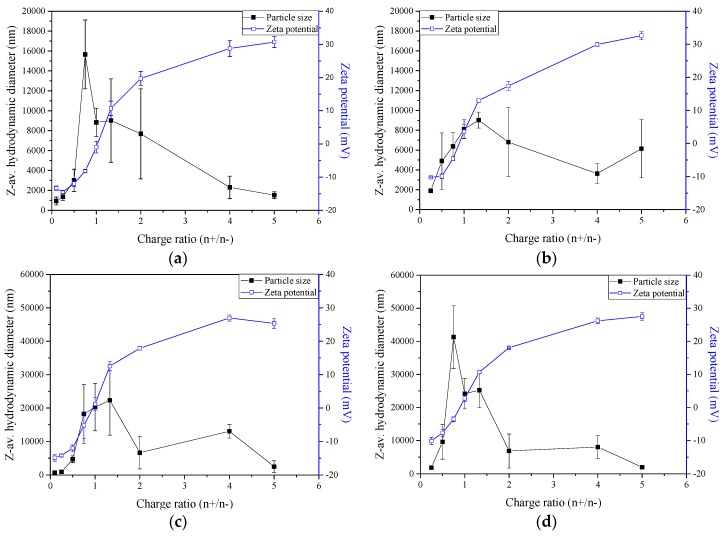
Variation of the Z-average hydrodynamic diameter and ζ-potential with charge ratio (n^+^/n^−^) of particles comprised by chitosan of different DA and pectin at pH 2.7 and 25 °C for systems with: (**a**) total charge 1.0 × 10^−6^ M, chitosan degree of acetylation (DA) 15.0%; (**b**) total charge 2.0 × 10^−6^ M, chitosan DA 15.0%; (**c**) total charge 1.0 × 10^−6^ M, chitosan DA 28.8; and (**d**) total charge 2.0 × 10^−6^ M, chitosan DA 28.8%.

It was expected that pectin’s carboxylate groups would interact electrostatically with oppositely charged amino functions of chitosan to form nano- and microparticles by polyelectrolyte complexation under controlled conditions to form stabilised colloidal particles. The ζ-potential (proportional to the surface charge density) values of the obtained particles are shown in Figure 2. Microparticles with size less than ~2500 nm were produced using chitosan with DA of 15.0% at 1.0 × 10^−6^ M total charge (Figure 2a) and charge ratios (n^+^/n^−^) 0.25 and 5.00, with a polydispersity index of 0.25 ± 0.06 and 0.40 ± 0.06, respectively, in good keeping with previously published values [24,33]. Based on the ζ-potential values (Figure 2a), we reasoned that the charge ratios (n^+^/n^−^) below 1.00 presented negative values, consistent with an excess of pectin in the nano- and microparticles system. At charge ratios (n^+^/n^−^) > 1.0, the resultant system exhibited a positive ζ-potential, in expected agreement with excess chitosan. Increasing the total charge (n^+^ + n^−^) from 1.0 × 10^−6^ to 2.0 × 10^−6^, produced larger particles at charge ratio (n^+^/n^−^) 0.25 and 5.00, with average diameters of ~2500 and ~5000 nm, respectively.

There was a very close similarity between the results observed for the systems with chitosan of DA 15.0% and those with chitosan of DA 28.8%. Microparticles with size less than ~1500 nm were obtained at charge ratio (n^+^/n^−^) 0.25 (Figure 2c,d). Negative ζ-potential values (Figure 2c,d) were evidenced for charge ratio (n^+^/n^−^) < 0.75, indicating an excess of pectin in the particle solution. As observed using chitosan with DA 15.0%, at charge ratio (n^+^/n^−^) > 1.0, the particles invariably attained a positive surface charge, associated with the stoichiometric surplus of chitosan. The ζ-potential dependence on charge ratio (n^+^/n^−^) obtained for the four analysed systems revealed that regardless of chitosan DA (either 15.0% or 28.8%) and the total concentration, the complexes were fully compensated (i.e., when the ζ-potential is zero) at charge ratio (n^+^/n^−^) very close to the stoichiometric point ((n^+^/n^−^) ~1.0). These results are in close agreement with those obtained in a previous study focused on chitosan (DA ~20%; molar mass ~230 kg·mol^−1^)/polyguluronate polyelectrolyte complexes [34]. In these systems, the equivalence point, as determined by conductimetric titrations, was also found at an equivalent charge ratio (n^+^/n^−^) ~1.0. From the above experiments, we selected four systems for subsequent studies of insulin loading, namely, the two chitosans (DA 15.0% and 28.8%), two charge ratios ((n^+^/n^−^) 0.25 and 5.00) and 1.0 × 10^−6^ M total charge.

### 2.4. Production Yield of Nano- and Microparticles

The production yield of nano- and microparticles loaded with insulin or unloaded (blanks) was calculated based on the complex mass obtained after lyophilisation and the initial mass of the pectin, chitosan, NaCl and insulin used (Table 1).

Inspection of Table 1 shows that the nano- and microparticles average hydrodynamic diameter was smaller after insulin loading, except for chitosan with a DA 28.8% at charge ratio (n^+^/n^−^) 5.00. This could indicate that the substitution of pectin by insulin at equivalent charge concentration considerably changed the particle size. An electrostatic interaction between the bioactive polypeptide and polymers (chitosan and pectin) could promote the small particles formation in solution, avoiding agglomerates of particles with greater size. The process of insulin addition to the nano- and microparticles was efficient, as there were no significant differences (*p* < 0.05) between the ζ-potential of the blank and insulin-loaded formulations.

Overall, higher production yields were obtained for the nano- and microparticles containing insulin than the blank ones. Particularly, in the formulations based on chitosan with DA 15.0% and charge ratio (n^+^/n^−^) 5.00, for which the increment in yield was ~100% (18.9% to 33.8% for blank and insulin-loaded systems, respectively). In the other systems studied, the insulin loading only moderately increased the yield (less than 10%). In general, our yield values agree with those of previous studies on chitosan-tripolyphosphate nano- and microparticles for drug delivery that found production yields ranging from 38–51% [35], 12–48% [36], and 24–84% [37].

### 2.5. Stability of Insulin-Loaded Nano- and Microparticles

**Figure 3:**
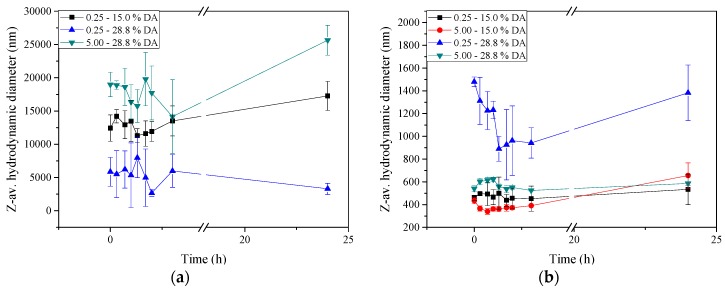

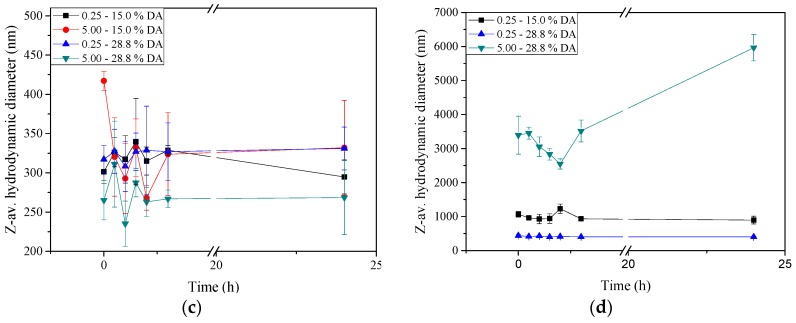
Evolution of the ζ-average hydrodynamic diameter of insulin-loaded particles comprised by chitosans of varying DA and pectin mixed at different charge ratio (n^+^/n^−^ as shown in labels) during incubation at 37 ± 1 °C for 24 h in: (**a**) 150 mM NaCl (pH 7.4); (**b**) MEM (minimal essential medium, pH 7.4); (**c**) SGF (simulated gastric fluid, pH 1.2) and (**d**) SIF (simulated intestinal fluid, pH 6.8).

The stability of insulin-loaded nano- and microparticles was evaluated by analysing the evolution of the particle size upon incubation in 150 mM NaCl (pH 7.4), minimal essential medium (MEM, pH 7.4), simulated gastric fluid (SGF, pH 1.2) and simulated intestinal fluid (SIF, pH 6.8), at 37 ± 1 °C for 24 h (Figure 3). As shown in Figure 3a, the particle size obtained from chitosan with DA 28.8% and charge ratio (n^+^/n^−^) 5.00 increased 4-fold after incubation in 150 mM NaCl for 24 h (from 5.0 to 20.0 µm). In contrast, the particle size obtained from chitosan with DA 28.8% and charge ratio (n^+^/n^−^) 0.25 (i.e., a surplus of negatively charged pectin) was notably smaller and remained stable at 24 h. Particles of smaller size tend to be more stable than larger particles against aggregation under physiological conditions, and are known to be also more effective to promote the absorption process of proteins through the intestinal epithelium [20,23]. Further, the particle size is an important characteristic to determine the absorption process, distribution, and in vivo performance of nanoparticles; it also influences the drug loading capacity and in vitro release characteristics of nanoparticles. In general, nanoparticles exhibit higher cellular uptake efficiency than do the larger size microparticles [14,21]. Nanoparticles with a particle size < 100 nm administered orally are known to be efficiently taken up in Peyer’s patches, and then absorbed into systemic circulation [38]. The evolution of the particle size in physiological conditions (150 mM NaCl and pH 7.4) is an indicator of the stability of colloidal particles during exposure to physiological isotonic conditions [39]. Interestingly, Lu et al. also observed that insulin-loaded polyelectrolyte complex nanoparticles of poly(glycerol methacrylate)s (cationic polymer) increased in size from 250 to 3000 nm upon increasing the NaCl concentration from 0.020 to 0.100 M [40]. They attributed this behaviour to the salt action, which could facilitate the polysaccharide complexation, thus resulting in larger particles size.

In general, the systems showed greater stability when incubated in MEM than in NaCl (Figure 3b). The average particle size in this instance was also significantly smaller (~300–~1600 nm). At 24 h incubation, the particle size remained below 700 nm, except for the formulation using chitosan with DA 28.8% and charge ratio (n^+^/n^−^) 0.25 that exhibited overall larger particle sizes after incubation. In previous studies, it was observed that chitosan-based nanocapsules obtained from chitosans of varying DA, were stable in cell culture medium, particularly those comprised by chitosan of high DA and low molecular weight [41]. We have argued that the hydrophilic nature of chitosan at the surface of these systems results in a stabilization mechanism based on short-range repulsive hydration forces. Russo et al. evaluated the stability of chitosan nanoparticles loaded with foscarnet, an antiviral agent, in phosphate buffered saline (PBS) at 37 °C and observed a gradual increase in particle size during the first 5 h [42]. To minimise this behaviour, the authors suggested adding a crosslinker (glutaraldehyde) to a particle preparation. This study also highlights the role of the polymers mobility on the overall colloidal stability at the surface of the particles. These effects seen previously by Russo et al. on the polymers mobility could also be observed in the chitosan/pectin complexes [42]. The behaviour of insulin-loaded nano- and microparticles was further evaluated in SGF (Figure 3c) and SIF (Figure 3d). The particle size upon incubation in SGF remained within 240–420 nm, and this persisted for 24 h.

Nano- and microparticles obtained at charge ratio (n^+^/n^−^) 0.25, independent of chitosan’s DA, showed particle diameters ~1000 nm after incubation in SIF. Increasing the charge ratio (n^+^/n^−^) from 0.25 to 5.00 (i.e., excess of positive charges from chitosan), the particles only remained stable for 3 h. Beyond this time, the particle size gradually increased upon incubation for up to 24 h. At pH close to neutrality, such as under SIF conditions, chitosan is practically non-ionised [27], which may explain the instability of the systems with excess chitosan. The increase in particle size can be attributed to aggregation of the particles rather than to swelling, as evidenced dynamic light scattering (DLS, results not shown).

Our results agree well with those of Bagre et al. who studied controlled delivery of enoxaparin from chitosan nanoparticles coated with alginate and found that the particles were not stable upon incubation in SIF (pH 7.4) [43]. They attributed this to the sensitivity of chitosan at the physiological pH of intestinal fluid after the dissolution of the alginate coat. Luo et al. evaluated the stability of lipid nanoparticles coated with chitosan intended for oral application, observing adequate results under acidic conditions [44]. The stability of insulin-loaded chitosan microspheres was evaluated under gastric pH, verifying that the insulin was protected from enzymatic degradation in this medium [45].

### 2.6. Insulin Encapsulation Efficiency (EE)

The EE of the insulin-loaded chitosan-pectin nano- and microparticles with different charge ratios (n^+^/n^−^, 0.25 and 5.00) and DA of chitosan (15.0% and 28.8%) are shown in Table 2. An EE of 34–37% of insulin was achieved for systems with charge ratio (n^+^/n^−^) 0.25. The EE was further improved (62%) for systems with charge ratio (n^+^/n^−^) 5.00, independent of DA of chitosan. We reason that the excess of positive chitosan charges considerably influenced the insulin loading of the nano- and microparticles.

Ideal nanoparticulate systems must have a high drug EE [46]. Different bioactive molecules, particularly biologics (e.g., enzymes, peptides, monoclonal antibodies), should maintain their bioactive conformation as to display the desired bioactive effects in vivo. Electrostatic interactions between the acidic groups of insulin and/or anionic polymers and amino groups of chitosan have important effects in the EE of insulin in chitosan-tripolyphosphate nanoparticles [47].

In previous studies focused on insulin-loaded nanoparticles comprised by polyelectrolyte complexation of chitosan and alginate and concomitantly by ionic gelation with tripolyphosphate, it was determined insulin encapsulation efficiency of 41–52% [48]. The chitosan/insulin mass ratio in these studies was fixed at 2.0 and in the present work varied between 0.4 and 0.5 (depending on DA of chitosan). However, in the mentioned studies they did not address the influence of the charge stoichiometry of chitosan to alginate, as we have done in the present work. It is clear from our results, that the composition of the system, determined by the proportional amount of chitosan is what governs the net amount of associated insulin. We propose that the association of insulin is mediated predominantly by electrostatic interactions between oppositely charged chitosan and insulin. The fact that the overall production yield was lower for the systems comprised by the chitosan of lower charge density (i.e., DA 28.8%) seems consistent with the idea that insulin is associated at the expense of the complexation of chitosan with pectin, as we have observed before in the chitosan–alginate–TPP (tripolyphosphate) nanoparticles [49].

Many studies have addressed the development of nanoparticles for oral delivery of insulin. Pan et al. found that chitosan nanoparticles can improve the intestinal absorption of insulin in alloxan-induced diabetic rats in up to 80% EE [49]. Bayat et al. reported that the insulin-loaded nanoparticles formed from chitosan and its derivatives (triethyl- and dimethylethylchitosan) had an EE between 84–90% [46]. Mukhopadhyay et al. produced self-assembled chitosan-insulin nanoparticles and obtained an insulin EE around 85% [16]. Al-Azi et al. found relatively lower values of EE, namely 5%, in insulin-loaded nanoparticles produced by complex coacervation between chitosan and pectin [19]. They attributed such low insulin association capacity to the low coacervation rate between the high molar mass polymers. Other studies have focused on the use of chitosan-based polyelectrolyte complex systems for drug delivery. Jardim et al. synthesised chitosan-chondroitin sulphate nanoparticles curcumin-loaded and found EE of curcumin ranged from 62.4 ± 0.61% to 68.3 ± 0.88% [5]. Zhang et al. prepared a polyelectrolyte complex based on chitosan and poly(2-acrylamide-2-methylpropanesulfonic acid) using a polymer-monomer pair reaction system and found the EE of doxorubicin ~61–64% [23].

### 2.7. Transmission Electron Microscopy

**Figure 4:**
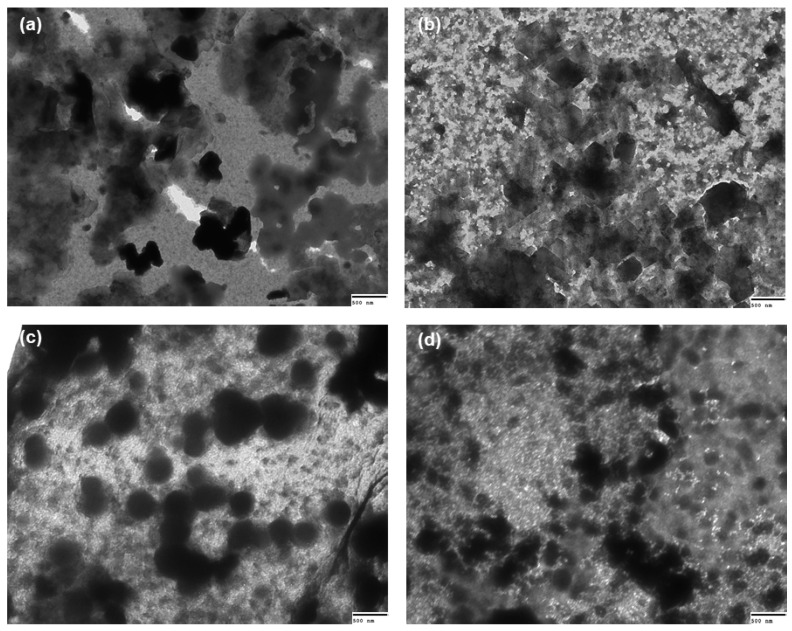
Representative transmission electron microscopy (TEM) images of (**a**) blank (unloaded) particles (chitosan DA 28.8% and charge ratio (n^+^/n^−^) 0.25); (**b**) Blank (unloaded) particles (chitosan DA 28.8% and charge ratio (n^+^/n^−^) 5.00); (**c**) insulin-loaded (chitosan DA 28.8% and charge ratio (n^+^/n^−^) 0.25); and (**d**) insulin-loaded (chitosan DA 28.8% and charge ratio (n^+^/n^−^) 5.00).

Images of nano- and microparticles non-loaded or insulin-loaded were recorded by transmission electron microscopy (TEM) and analysed in terms of morphology and surface topology. Representative images of the systems formulated using chitosan DA 28.8% at two different charge ratios (n^+^/n^−^, 0.25 and 5.00) are shown in Figure 4. Very similar micrographs were recorded for systems comprised by chitosan DA 15.0% (results not shown). It was interesting to notice that the systems loaded with insulin revealed a more spherical morphology (Figure 4c,d) than the corresponding blank ones, independently of the charge ratio (n^+^/n^−^). Insulin-loaded chitosan particles presenting spherical shape and smooth surface have also been found in previous studies [16,48]. The range of particle size observed in the TEM images in all cases seems smaller compared to those obtained from DLS analysis. This could be the expected consequence of the larger abundance by number of smaller particles over the larger ones, hence these are more likely to be better imaged in suitable windows of the grid during TEM. Also, the hydrodynamic diameter of freshly prepared nano- and microparticles measured by DLS may be expanded due to swelling, whereas such effect is likely to be abrogated during the preparation of the specimens for TEM [20].

### 2.8. Cytotoxicity (MTT (3-(4,5-Dimethylthiazolyl-2)-2,5-diphenyltetrazolium bromide) Assay)

Evaluation of the cytocompatibility of any new drug delivery biomaterial is a crucial step in its development, as it offers a first in vitro proof-of-concept of its biocompatibility of [50]. The MTT assay is the most common method to assess the effect of either biomaterials or bioactive compounds using mammalian cell lines in culture [51,52]. This also enables comparisons with previous studies.

The reduction of MTT, a yellow tetrazolium salt, occurs by metabolically competent mitochondria. After reduction, a violet formazan dye is formed, and the metabolic competence (assumed to be directly proportional to the cell viability) can be quantified spectrophotometrically by comparing the results to the corresponding positive and negative controls [53]. Caco-2 cells are frequently used as representative cells of the human intestine due to their capacity to spontaneously differentiate after 3–4 weeks into highly polymerised phenotypes [54] with functional tight junctions in porous membranes [55].

**Figure 5:**
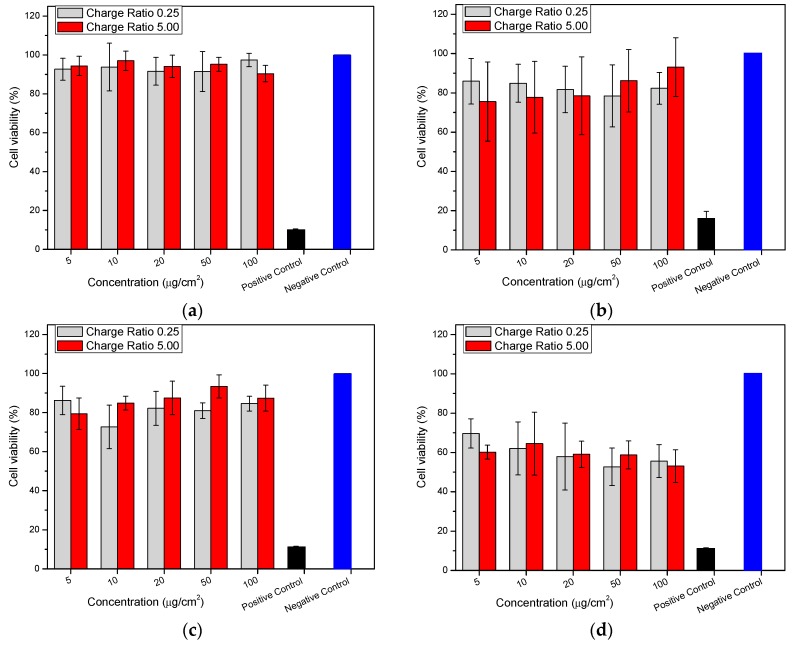
Cell viability (MTT (3-(4,5-dimethylthiazolyl-2)-2,5-diphenyltetrazolium bromide) assay) of Caco-2 cells after treatment (4 h at 37 ± 0.1 °C) varying concentrations of nano- and microparticles at different charge ratio (n^+^/n^−^) (as in figure legends). (**a**) Blank (unloaded) particles, chitosan DA 15.0%; (**b**) Insulin-loaded, chitosan DA 15.0%; (**c**) Blank (unloaded) particles, chitosan DA 28.8% and (**d**) Insulin-loaded, chitosan DA 28.8% (values represent average and standard deviations of three biological replicates).

In this study, the in vitro cytotoxicity of the chitosan-pectin nano- and microparticles at different concentrations was evaluated by MTT tests (Figure 5). Blank nano- and microparticles showed cell viability values above 90% (Figure 5a) and 80% (Figure 5c), irrespective of the chitosan’s DA and the chitosan–pectin charge ratio (n^+^/n^−^). No dose-response effect was observed upon treating the cells with particle concentrations from 5–100 µg·cm^−2^. Cell viability did not decrease even after incubation at 37 °C for 4 h. In all the blank formulations, the particles neither showed any particular cytotoxicity nor influenced the normal growth of Caco-2 cells within the dose range studied (≤100 µg·cm^−2^). However, the insulin-loaded particles decreased the cell viability, which was influenced by the chitosan’s DA (Figure 5b,d). While moderate decreases (75–95%) in cell viability were observed for the systems using chitosan with DA 15% (Figure 5c), the decrease was notably more evident (55–70%) in the treatments comprising chitosan DA 28.8% (Figure 5d) at both the chitosan/pectin equivalent charge ratios (n^+^/n^−^) studied.

The significantly accentuated decrease in cell viability observed for the insulin-loaded systems using chitosan with DA 28.8% compared to chitosan with DA 15.0%, reveals that the DA of chitosan was an important characteristic that influenced the cytocompatibility of these systems. An overall lower cell viability and a dose-response at increasing concentrations (from 5.0 to 100 µg·cm^−2^) were observed for the particles using chitosan with DA 28.8% than for those comprising chitosan of DA 15.0%. This could be attributed to the role of insulin as an endogenous hormone that can interfere with normal cell metabolism [50]. The differences in cell viability associated with the DA of chitosan could stem from the greater intracellular delivery achieved by the particles using chitosan with DA 28.8% than the more highly-charged chitosan.

Previous studies have addressed the cytocompatibility of chitosan-based nanoparticle formulations. Loretz and Bernköp-Schnurch proposed that in general, the decrease in Caco-2 cell viability upon treatment with chitosan (low viscosity and DA 19.0%) could result from electrostatic interactions between chitosan nanoparticles and the cell membrane [56]. This was confirmed by Soliman et al. who reported a simple and bioinspired nanoparticulate system by preparing hydrocaffeic acid-chitosan conjugates loaded with bovine serum albumin [39]. They found accentuated cell viability decreases in the presence of the drug. Biswas et al. developed and characterized sodium alginate coated chitosan (42, 74 and 106 kDa) nanoparticles, in which the measles antigen was entrapped and found no significant difference in cell viability [57]. In another study, Jia et al. studied the transport of chitosan (14% DA) nanoparticles across Caco-2 cell monolayers [58]. They found that the cytotoxicity was mainly influenced by the molar mass of chitosan (200 kDa) and its cationic charge density, promoting a high degree of interaction between chitosan and the cell membrane. Sadeghi et al. evaluated the permeation of insulin-chitosan nanoparticles through a Caco-2 cell monolayer using chitosan and its derivatives (trimethyl-, dimethylethyl-, diethylmethyl- and triethylchitosan) and concluded that the polymers did not exert any cytotoxicity (less than 1.0% dead cells) [59]. Similar results were reported by Zhang and Zhao who investigated chitosan nanoparticles and found cell viability values above 84.0% [36].

### 2.9. Insulin Release: In Vitro Tests

**Figure 6:**
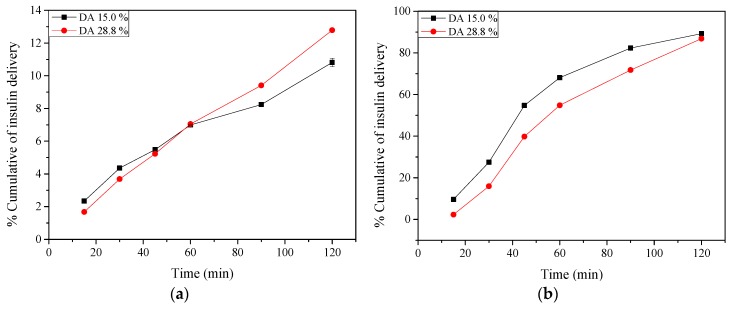
Percentage cumulative of insulin release from nano- and microparticles prepared using chitosan of different DA and pectin at charge ratio (n^+^/n^−^) 5.00 during incubation for 120 min at 37 ± 0.1 °C in: (**a**) Simulated gastric fluid (pH 1.2) and (**b**) Simulated intestinal fluid (pH 6.8).

Insulin release from nano- and microparticles prepared using different chitosans (DA 15.0% and 28.8%) at charge ratio (n^+^/n^−^) 5.00 (selected from the higher EE values) was studied in SGF (pH 1.2) (Figure 6a) and SIF (pH 6.8) (Figure 6b) for 120 min, respectively. The chitosan DA did not influence the process of insulin release, presenting similar behaviour under both conditions. In SGF, initially, a small amount of insulin was released (~2%) (Figure 6a), followed by a controlled release that after 120 min did not exceed ~13%. Note that the overall release of insulin in SGF (pH 1.2) was much lower than that in simulated intestinal fluid (pH 6.8). This is believed to be caused by the weak electrostatic interaction between the insulin, bearing a highly positive net charge, and the polyelectrolytes on the surface. Further, insulin released from nano- and microparticles comprising the two chitosans (DA 15.0% and 28.8%) in conditions that simulated the human intestine (pH 6.8) was controlled (Figure 6b) and attained a maximum of 89.0% after 120 min.

The insulin delivery from nano- and microparticles carried out in SIF (Figure 6b) showed a controlled and slightly prolonged insulin release, amounting to 87.0–89.0% of its initial amount. This could be due to the strong interaction between the alkaline solvent and positively charged chitosan shell, facilitating the penetration of the solvent towards the pectin core. In contrast, the positive charges from chitosan and the tight pectin network could help to retain the insulin at pH 1.2 and protect the encapsulated insulin against proteolytic degradation in the stomach [16]. The behaviour of insulin release under acidic conditions is in accordance with previous studies [21,43,60]. However, the rapid insulin delivery at pH 6.8 was an unexpected result.

For the measles antigen entrapped in coated and uncoated chitosan nanoparticles, Bagre et al. found that the antigen was rapidly delivered (40.0% in 2 h) in SGF using the uncoated nanoparticles, which was associated to the high solubility of chitosan at low pH [43]. Biswas et al. observed rapid antigen delivery (~20.0%) from chitosan nanoparticles in PBS (pH 7.4) at 3 h [57]. The authors attributed this to a weak ionic interaction between the bioactive and chitosan, which could be easily desorbed in an ionic environment. Mukhopadhyay et al. prepared chitosan-alginate nanoparticles to incorporate insulin and evaluated the delivery process in gastric (pH 1.2) and intestinal (pH 6.8 and 7.4) media [21]. Rapid delivery of insulin (26.7%) was observed in the gastric medium after 2 h, which was attributed to weak interactions between insulin and the poly-electrolytes. In SIF, however, a slow and modulated delivery of insulin (79.0–84.0%) was found at 24 h.

## 3. Materials and Methods

### 3.1. Materials

Two high-purity, research grade chitosan samples (chitosan 85/5 Nr. 23500, lot 212-300811-03, herein referred to as sample “HMC^+^ 15”, and chitosan 70/5 Nr. 23200, lot 212-111111-03, herein referred to as sample “HMC^+^ 28.8”), were purchased from Heppe Medical Chitosan GmbH (Halle, Germany) and used as received; pectin (from citrus peel) with a high DE (GENU^®^ 105 rapid set, lot LI03024) was from CPKelco (Limeira, Brazil); and recombinant human insulin (molar mass of 5.7 kDa, lot 11D762J) was from SAFC (Lenexa, KS, USA). All reagents were of analytical grade. Ultrapure MilliQ water was used throughout.

### 3.2. Methods

#### 3.2.1. Determination of the Degree of Acetylation of Chitosan

The DA of the chitosan samples was determined by ^1^H-NMR spectroscopy, per the procedure of Lavertu et al. [61], using a DRX 500 spectrometer (Bruker, Fallanden, Switzerland). Briefly, chitosan powder (5.0 mg) was dissolved in 1.0 mL HCl (37%), frozen at −20 °C for 24 h, then lyophilised for 12 h. After, the sample was dissolved in 1.0 mL of deuterated water (D_2_O). The spectra were acquired at 25 °C. The DA (%) was calculated using integrals of the peak of the proton of deacetylated monomer (H_1_D) and of the peak of the three protons of the acetyl group (H-Ac), i.e., DA (%) = 100 − [[H_1_D/(H_1_D + H-Ac/3)] × 100].

#### 3.2.2. Determination of Intrinsic Viscosity ([*η*]) and Molar Mass Estimation of Chitosans

The [*η*] of the chitosan samples was determined according to Rinaudo et al. [62]. Briefly, HMC^+^ 15 chitosan solutions (1.25, 1.50, 1.75, 2.00 and 2.50 mg·mL^−1^) and HMC^+^ 28.8 solutions (2.00, 2.50, 3.00, 4.00 and 5.00 mg·mL^−1^) were prepared in 0.3 M AcOH/0.2 M AcONa. The relative viscosity was measured using a model AMVn automatic capillary rolling ball microviscometer (Anton Paar, Scharnhausen, Germany) at 25 ± 0.2 °C (four determinations per concentration). The [*η*] (mL^·^g^−1^) was estimated by extrapolation and averaging from the Huggins (Equation (1)), Kraemer (Equation (2)) and Solomon-Ciuta equations (Equation (3)):

(1) $$\frac{\eta_{sp}}{C}=\left[\eta \right]+\;{\left[\eta \right]}^{2}k_{H}C$$

where $\frac{\eta_{sp}}{C}$ is the reduced viscosity (mL·g^−1^), $k_{H}$ is the Huggins’ coefficient and *C* is the polymer concentration (g·mL^−1^).

(2) $$\ln({\left[\eta \right]}_{rel})/C=\left[\eta \right]+\;{\left[\eta \right]}^{2}k_{K}C$$

where $\ln({\left[\eta \right]}_{rel})/C$ is the inherent viscosity, $k_{K}$ is the Kraemer’ constant and *C* is the polymer concentration (g·mL^−1^).

(3) $$\left[\eta \right]={\left[2\;\left(\eta_{sp}-\ln{\;\eta }_{red}\right)\right]}^{1/2}/C$$

where $\eta_{sp}$ is the specific viscosity, $\eta_{red}$ is the reduced viscosity and *C* is the polymer concentration (g·mL^−1^).

The viscosimetric mean average molar mass, *M*, was then estimated from the [*η*] value using Mark-Houwink-Sakurada relationship (Equation (4)):

(4) $$\left[\eta \right]=KM^{\alpha }$$

where, *K* = 7.4 × 10^−2^ and 7.6 × 10^−2^ (mL·g^−1^) for HMC^+^ 28.8 and HMC^+^ 15, respectively, and *α* = 0.76 for both [60].

#### 3.2.3. Determination of the Degree of Esterification of Pectin

In accordance with Rosenbohm et al. 5.0 mg of pectin was dissolved in 0.8 mL of D_2_O under magnetic stirring at 55–60 °C for 1 h, frozen at −20 °C for 24 h, lyophilised and then 1.0 mL of D_2_O added and the mixture stirred until fully dissolved [63]. The ^1^H-NMR spectra were acquired at 25 °C on a DRX 500 model spectrometer (Bruker). The DE (%) is defined as the number of ester groups compared to the total number of carboxylic acid and ester groups. The DE was determined by comparing integrals of H-5 adjacent to esters (I_COOMe_) to the sum of the integrals of H-5 adjacent to esters (I_COOMe_) and H-5 adjacent to carboxylates (I_COO−_). Due to the proximity (or overlap) of the signals for H-1 and H-5_COOMe_, it was only possible to determine the combined integrals for H-1 and H-5_COOMe_ (I_H1_+I_COOMe_). This value can be introduced into the equation for the DE because the total number of the H-5 protons is equal to the sum of the anomeric H-1 protons: I_COOMe_ + I_COO−_ = I_H1_. The DE can be determined from DE (%) = [(I_COOMe +_ I_H1_) – I_COO−_)/[(I_COOMe +_ I_H1_) + I_COO−_)] × 100.

#### 3.2.4. Determination of Intrinsic Viscosity ([*η*]) and Molar Mass Estimation of Pectin

The [*η*] (mL·g^−1^) of pectin solutions prepared in 100 mM NaCl (0.125, 0.250, 0.500, 1.000, 1.500 and 2.000 mg·mL^−1^) was measured as described in Section 3.2.2. The mean average molar mass, *M*, was estimated from the [*η*] values using the Mark−Houwink equation (Equation (4)). The constants used for pectin with a DE between 30.0–95.0%, were *K* = 9.55 × 10^−2^ (mL·g^−1^) and *α* = 0.73 [64].

#### 3.2.5. Preparation of Nano- and Microparticles

##### Chitosan Solutions

Chitosan solutions (5.0 mg·mL^−1^, *w*/*v*) were prepared in 5% stoichiometric excess of HCl and in 100 mM NaCl by continuous magnetic stirring at room temperature (25 ± 1 °C) for 14 h, followed by filtration through 5.0 µm membranes (EMD Millipore, Burlington, MA, USA). The ζ-potential of the chitosan solutions (5.0 mg·mL^−1^, *w*/*v*) was measured by mixed laser Doppler velocimetry and phase analysis light scattering (M3–PALS). A Malvern Zetasizer Nano ZS (Malvern Instruments Ltd., Worcestershire, UK) fitted with a red laser light (λ = 632.8 nm) was used. The ζ-potential was measured at pre-determined intervals over the pH range 1.0–8.0, to assess the best conditions to obtain nano- and microparticles. Triplicate measurements were performed.

##### Pectin Solutions

Pectin solutions (5.0 mg·mL^−1^, *w*/*v*) were prepared in 100 mM NaCl by continuous magnetic stirring at 50 ± 1 °C for 1 h (to ensure the pectin would be solubilised) and then at room temperature (25 ± 1 °C) for 13 h, followed by filtration through 5.0 µm membranes (EMD Millipore). The ζ-potentials of the pectin solutions (5.0 mg·mL^−1^, *w*/*v*) was measured as described in the previous section.

##### Pectin Purification

This was done according to Bernabé et al. [65]. Briefly, pectin (2.0 g·L^−1^) was dissolved in 50 mM NaCl by continuous magnetic stirring at 50 ± 1 °C for 1 h and then, at room temperature (25 ± 1 °C) for 13 h. This solution was filtered successively through glass wool, sintered glass filters (pore diameters of 80.0, 60.0, 40.0 and <10.0 µm) and membranes (5.0, 1.2, 0.8, 0.45 and 0.2 μm, EMD Millipore). The polysaccharide was precipitated by gradual addition of ethanol until a final 80.0% (*v*/*v*) alcohol concentration was obtained. The precipitate was removed by centrifugation (Sorvall, R-5 plus model, Langenselbold, Germany) at 7000 rpm, 10 °C for 30 min. The solid was washed with ethanol/water mixtures (70/30, 80/20, 90/10 and 100/0) for 5 min each. The purified polymer was dried at room temperature (25 ± 1 °C) for 48 h.

#### 3.2.6. Preparation of Nano- and Microparticles

Nano- and microparticles were prepared by electrostatic self-assembly according to the method reported by Fuenzalida et al. [66]. Briefly, chitosan-pectin mixed systems were prepared at 0.10, 0.25, 0.50, 0.75, 1.00, 1.33, 2.00, 4.00 and 5.00 charge equivalent ratios (equiv. × L^−1^) (n^+^/n^−^), and 1.0 × 10^−6^ and 2.0 × 10^−6^ M total charge (n^+^ + n^−^). The formation and optimal conditions to obtain nano- and micro-particles were screened by preparing the mixtures in a 96-well microplate (final volume 250 µL). An appropriate aliquot of chitosan solution (5.0 mg·mL^−1^, *w*/*v*) was added into each microwell. An aliquot of mixed pectin (5.0 mg·mL^−1^, *w*/*v*) and NaCl (100 mM) was added into the chitosan solution and mixed thoroughly by flushing the mixture in and out of the pipette tip. The conversion of the final solution (chitosan + pectin + NaCl) from a clear/limpid to turbid/opalescent appearance, was taken as preliminary evidence of nanoparticle formation. When necessary, isolated nano- and micro-particles solutions were obtained by centrifugation (10,000× *g*, 20 °C for 40 min) using Eppendorf tubes containing 15 µL of glycerol. The pellets were re-suspended in 100 µL of 100 mM NaCl.

Insulin-loaded nano- and microparticles were prepared by considering the best charge ratios (n^+^/n^−^ 0.25 and 5.00) and total equiv. charge (1.0 × 10^−6^ M) results. A portion (30.0%) of the charges of one polymer was substituted with insulin charges. Insulin (5.0 mg·mL^−1^) was dissolved in one of the following solutions: 10 mM NaOH/100 mM NaCl (charge ratio (n^+^/n^−^) 0.25) or 100 mM NaCl, pH adjusted to 2.70 (charge ratio (n^+^/n^−^) 5.00).

#### 3.2.7. Zeta-potential and Particle Size Determination of Nano- and Microparticles

Nano- and microparticles suspensions were prepared at charge ratios (n^+^/n^−^) 0.25 and 5.00, 1.0 × 10^−6^ total charge and in 100 mM NaCl solution. The particle size distribution was obtained by dynamic light scattering with non-invasive back scattering (DLS-NIBS) at 173° with automatic gain using a Malvern Zetasizer Nano ZS. The ζ-potential was measured as mentioned previously. All measurements were recorded in triplicate at 25 ± 2 °C.

#### 3.2.8. Production Yield

Nano- and microparticles, produced with and without insulin, were centrifuged (20 °C, 10,000× *g*, 40 min). The supernatant was removed, and the pellets were frozen at −20 °C and lyophilised for 48 h. The production yield of the particles was calculated from the masses of chitosan, pectin, insulin (or not) and NaCl used in the initial suspension preparation and the mass of the pellet formed (Equation (5)):

(5) $$Yield\;\left(\%\right)=\frac{M_{pellet}}{\left(M_{c}+M_{p}+M_{ins}+M_{NaCl}\right)}\times 100$$

where, $M_{pellet}$ is the mass of particles obtained after lyophilised process and $M_{c}$, $M_{p}$, $M_{ins}$ and $M_{NaCl}$ are the respective initial masses of chitosan, pectin, insulin and sodium chloride used for nano- and microparticles formation.

#### 3.2.9. Stability Tests

The colloidal stability of the systems was assessed as described elsewhere [67]. Briefly, the isolated nano- and microparticle solutions were incubated in 150 mM NaCl (adjusted pH to 7.4 using 0.1 M NaOH and 0.1 M HCl), MEM (pH 7.4), SGF (pH 1.2) and SIF (pH 6.8) in a microtiter plate incubator (Titramax model, Heidolph, Schwabach, Germany) at 37 ± 0.1 °C. At pre-determined times (0, 20, 40 60, 90, 120, and 180 min, and 24 h) the particle size distribution was measured by DLS-NIBS as described in Section 3.2.7.

*SGF solution.* NaCl (0.4 g) was solubilised in 180 mL of deionised water and adjusted to pH 1.2 using 0.1 M NaOH and 0.1 M HCl. After, the final volume was made up to 200 mL. The pH was 1.2.

*SIF solution.* H_2_KPO_4_ (1.36 g) was dissolved in 50 mL of deionised water. Separately, 15.4 mL of 0.2 M NaOH and 100 mL of deionised water were combined. The solutions were homogenised together and adjusted to pH 6.8 using 0.1 M NaOH and 0.1 M HCl. After, the final volume was made up to 200 mL. The pH was 6.8. Both the SGF and SIF solutions were prepared according to the United States Pharmacopeia XIX.

#### 3.2.10. Insulin Encapsulation Efficiency

The insulin EE in the different systems was determined using the protocol described by Marschütz and Bernkop-Schnürch [68], as modified by Krauland and Alonso [69]. The supernatant obtained after isolation was filtered (0.22 µm, Millipore) and then a 5.0 µL aliquot injected into a HPLC system (model X-LC, Jasco, Easton, PA, USA) equipped with a peptide C18 reverse phase column (Aeris wide pore XB-C18, 3.6 µm 150 × 2.1 mm, Phenomenex, Aschaffenburg, Germany) at room temperature (25 °C). Gradient elution was performed as follows: 0.3 mL·min^−1^ flow rate for the first 5 min; linear gradient from 70.0% A/30.0% B to 39.0% A/61.0% B (eluent A: 0.1% trifluoracetic acid in water; eluent B: acetonitrile). Insulin and/or degradation products were detected at λ = 220 nm with a UV-Vis detector. Insulin concentrations were quantified from integrated peak areas and calculated using a calibration curve. The EE of insulin was calculated from the following equation (Equation (6)):

(6) $$Encapsulation\;efficiency\;\left(EE,\;\%\right)=\frac{Total\;{}_{Ins}-Free_{Ins}}{Total_{Ins}}\times 100$$

where, $Total{\;}_{Ins}$ is the total quantity of insulin used to load at nano- and microparticles and $Free_{Ins}$ is the quantity of insulin not associated to the nano- and microparticles.

#### 3.2.11. Transmission Electron Microscopy

Nano- and microparticles unloaded and loaded-insulin formulated using chitosan DA 28.8% at two different charge ratios (n^+^/n^−^) (0.25 and 5.00) were visualized by TEM. To this end, 20μL of freshly prepared samples were mixed with 20μL of 1% (*w*/*v*) uranyl acetate for negative staining. Afterwards 8μL of samples were deposited onto a copper grid covered with Formvar^®^ film. Excess liquid was blotted using filter paper. Images were captured using a JEM-1400 TEM (JEOL, Peabody, MA, USA) operating at 100 kV. Images were processed on an AMT 1k CCD (AMT, Woburn, MA, USA) using AMTV602 software (AMT, Woburn, MA, USA).

#### 3.2.12. Cytotoxicity (MTT Assay)

##### Cell Culture

Caco-2-cells were cultured in 75 cm² flasks using MEM supplemented with 10% foetal bovine serum, 1% l-glutamine (200 mM) and 1% penicillin-streptomycin (10,000 units penicillin, 10,000 units streptomycin in 0.9% NaCl). The cultures were maintained in a humid atmosphere at 37 °C with 5% CO_2_ (Sanyo MCO-19AIC, Panasonic Biomedical Sales Europe BV, AZ, Etten-Leur, The Netherlands). Cells from passages 43, 44, 46 and 50 were used for all experiments, which were carried out as independent triplicates on different days. After reaching microscopic confluence, the cells were washed with 10 mL PBS and trypsinised with 10 mL of 0.05% trypsin in EDTA (1×) buffer. After detachment, 10 mL of MEM was added to the trypsin buffer. The cell suspension was centrifuged at 1000 rpm for 5 min (Rotina 420 R, Andreas Hettich GmbH, Tuttlingen, Germany). The excess medium was removed, and the cell pellet was resuspended in 1 mL MEM. A 10 µL aliquot of the cell suspension was diluted with 90 µL trypan blue, and the number of cells was counted with an improved Neubauer chamber before seeding. The cells were sub-cultured by splitting at a 1:10 ratio.

##### MTT Assay

This assay was used to evaluate the cytotoxicity of the formulations and components. Briefly, 100 µL of cell suspension was transferred to each well of a 96-well tissue culture plate (~10^4^ cells per well or ~10^5^ cells × mL^−1^) and allowed to attach for 24 h. The cells were washed twice with supplement-free MEM before the sample was added and the cells were then incubated for 3 h. Nano- and micro-particles prepared in different charge ratio (n^+^/n^−^ 0.25 and 5.00) using chitosan with different DA (15.0% and 28.8%) were isolated as detailed previously and re-suspended in 100 µL of MEM. Aliquots (100 µL) were then placed into each well and incubated for 4 h. The samples were removed and replaced with 100 µL supplement-free MEM. A MTT solution in PBS with thiazolyl blue tetrazolium bromide (25 µL, 5.0 mg·mL^−1^) was added to each well. After 4 h, the medium was again removed, and DMSO was added to dissolve the MTT formazan crystals. The plates were protected against light during agitation at 100 rpm for 15 min in an orbital shaker (KS 4000i control model, IKA, Staufen, Germany). After, the absorbance was measured at λ = 570 nm with a microplate reader (Safire, Tecan AG, Salzburg, Austria). Positive (cell death using 100 μL of Triton X 4.0% (*w*/*v*)) and negative (cell death using 100 μL of MEM without particles) controls were used to determine the relative cell viability. Each concentration was studied in eight microplates, and the biological experiments were carried out in triplicate [70].

#### 3.2.13. Insulin Release: In Vitro Tests

Insulin release assays were performed according to Bagre et al. [43]. Chitosan of different DA (15.0% and 28.8%) and pectin solutions were prepared to obtain isolated nano- and microparticles loaded with insulin at charge ratio (n^+^/n^−^) 5.0 and total charge of 1.0 × 10^−6^. The nano- and microparticle dispersions (100 µL) were placed into Eppendorf tubes containing 900 µL of SIF or SGF and maintained in an incubator (Titramax model, Heidolph) at 37 ± 1 °C. Aliquots were removed at pre-determined times (0, 15, 30, 45, 60, 90 and 120 min) and the insulin content was determined as described in Section 3.2.10. The insulin release experiments were carried out in triplicate.

### 3.3. Statistical Analysis

The data were appraised using the Statistica program version 7.0 (Statistica Inc., Palo Alto, CA, USA). Differences between the means were detected by Tukey’s multiple comparisons test.

## 4. Conclusions

Pectin–chitosan nano- and microparticles were obtained by electrostatic self-assembly. The small particle sizes, ranging from 240–1900 nm were amenable to encapsulate insulin. The optimal charge ratio (n^+^/n^−^) for nano- and microparticles formation were 0.25 (excess of pectin charges) and 5.00 (excess of chitosan charges), which produced the highest product yield (maximum 33.8%). Insulin was efficiently encapsulated (from 34.0–62.0%) into the particles due to a strong interaction between the oppositely charged polysaccharides. Controlled insulin release occurred under SIF (pH 6.8) conditions, and less than 13.0% was released in SGF (pH 1.2, 2 h). The non-cytotoxicity (using chitosan with DA 15.0%) of the proposed system seems to be advantageous due to its simple manufacture, offering an alternative route for oral insulin delivery with good stability under acidic (stomach) and basic (intestinal) conditions.

## Figures and Tables

**Table 1:** Particle size, ζ-potential and production yield of chitosan-pectin unloaded or loaded with insulin prepared at different charge ratios (n^+^/n^−^) and comprising chitosans of varying degree of acetylation.

Analysis	Insulin	Chitosan DA 15.0%		Chitosan DA 28.8%	
		Charge Ratio (n^+^/n^−^)		Charge Ratio (n^+^/n^−^)	
		0.25	5.00	0.25	5.00
Particles size (d, nm)	Blank	2530 ± 384 ^aA^	2618 ± 175 ^aA^	1875 ± 135 ^aA^	2011 ± 266 ^aA^
	Loaded	1351 ± 384 ^aB^	1522 ± 346 ^aB^	964 ± 32 ^aB^	2510 ± 107 ^bB^
ζ-potential (mV)	Blank	−22.5 ± 0.8 ^aA^	+35.0 ± 1.4 ^bA^	−23.4 ± 0.9 ^aA^	+27.2 ± 1.4 ^bA^
	Loaded	−22.5 ± 2.5 ^aA^	+33.2 ± 2.3 ^bA^	−22.4 ± 2.4 ^aA^	+28.6 ± 2.0 ^bA^
Production yield (%)	Blank	23.8 ± 1.1 ^aA^	18.9 ± 3.0 ^bA^	22.6 ± 0.6 ^aA^	23.7 ± 3.3 ^aA^
	Loaded	27.2 ± 3.9 ^aA^	33.8 ± 4.3 ^aB^	24.3 ± 2.0 ^aA^	22.4 ± 2.0 ^aA^

^a,b^: Average of different letters in the same line for the same analysis and type of chitosan differ statistically (*p* < 0.05) by Tukey test. ^A,B^: Average of different letters in the same column for the same analysis and type of chitosan differ statistically (*p* < 0.05) by Tukey test.

**Table 2:** Encapsulation efficiency (EE) of insulin at the nano- and microparticles prepared in different charge ratios (n^+^/n^−^) and degree of acetylation (DA) of chitosan.

Formulation	Charge Ratio (n^+^/n^−^)	DA (%)	EE (%)
1	0.25	15.0	36.6 ± 6.6 ^a^
2	5.00	15.0	62.2 ± 3.1 ^b^
3	0.25	28.8	34.2 ± 8.1 ^a^
4	5.00	28.8	61.9 ± 0.5 ^b^

^a,b^: Average of different letters in the same column differ statistically (*p* < 0.05) by Tukey’s test.
